# Damaged-self recognition in common bean (*Phaseolus vulgaris*) shows taxonomic specificity and triggers signaling via reactive oxygen species (ROS)

**DOI:** 10.3389/fpls.2014.00585

**Published:** 2014-10-31

**Authors:** Dalia Duran-Flores, Martin Heil

**Affiliations:** Laboratory of Plant Ecology, Departamento de Ingeniería Genética, Centro de Investigación y de Estudios Avanzados del Instituto Politécnico Nacional Unidad IrapuatoIrapuato, México

**Keywords:** extrafloral nectar, danger signal, damage-associated molecular pattern, induced defense, induced resistance, plant pathogen, *Pseudomonas syringae*, wound response

## Abstract

Plants require reliable mechanisms to detect injury. Danger signals or “damage-associated molecular patterns” (DAMPs) are released from stressed host cells and allow injury detection independently of enemy-derived molecules. We studied the response of common bean (*Phaseolus vulgaris*) to the application of leaf homogenate as a source of DAMPs and measured the production of reactive oxygen species (ROS) as an early response and the secretion of extrafloral nectar (EFN) as a jasmonic acid (JA)-dependent late response. We observed a strong taxonomic signal in the response to different leaf homogenates. ROS formation and EFN secretion were highly correlated and responded most strongly to leaf homogenates produced using the same cultivar or closely related accessions, less to a distantly related cultivar of common bean or each of the two congeneric species, *P. lunatus* and *P. coccineus*, and not at all to homogenates prepared from species in different genera, not even when using other Fabaceae. Interestingly, leaf homogenates also reduced the infection by the bacterial pathogen, *Pseudomonas syringae*, when they were applied directly before challenging, although the same homogenates exhibited no direct *in vitro* inhibitory effect in the bacterium. We conclude that ROS signaling is associated to the induction of EFN secretion and that the specific blend of DAMPs that are released from damaged cells allows the plant to distinguish the “damaged-self” from the damaged “non-self.” The very early responses of plants to DAMPs can trigger resistance to both, herbivores and pathogens, which should be adaptive because injury facilitates infection, independently of its causal reason.

## INTRODUCTION

Like all multicellular organisms, plants require efficient mechanisms to detect injury. Specificity in the resistance to specialist herbivores and pathogens can be adaptive, because it allows for the synthesis of defensive compounds that are highly active against the current attacker. Specific responses can be triggered by the recognition of herbivore-associated molecular patterns (HAMPs; Wu and Baldwin 2010) or microbe- (or pathogen-) associated molecular patterns (MAMPs/PAMPs) in plants (Jones and Dangl, 2006). Common HAMPs are fatty acid-amino acid conjugates that stem from the saliva of insects or compounds in insect oviposition fluids (Wu and Baldwin, 2010). Common MAMPs are flagellin, chitin, and other molecules that are not produced by plants and that indicate the presence of enemies on or in a plant. Effector molecules that are secreted by adapted pathogens to overcome the plant resistance response can also be perceived as strain-specific PAMPs and then cause effector-triggered immunity (Jones and Dangl, 2006). Most of these molecules are recognized by pattern recognition receptors (PRRs), which induce a signaling cascade that normally consists of Ca^2+^-influxes and the corresponding membrane depolarization events, the generation of reactive oxygen species (ROS) such as the superoxide anion (O_2_^-^), hydrogen peroxide (H_2_O_2_) or the hydroxyl radical (⋅OH), and downstream signaling via mitogen-associated protein kinase (MAPK) cascades (Wu and Baldwin, 2010).

However, successfully dealing with injury requires multiple countermeasures that are independent of the detailed nature of the injury-causing agent. For example, wounds must be sealed to avoid desiccation and even so, they normally will be used by pathogens to enter the wounded tissue (Komarova et al., 2014). Moreover, induced plant responses that are completely based on the recognition of specific HAMPs or PAMPs would always be in danger to be overcome by co-evolving enemies, which change the molecular structure of the target molecules of plant receptors and thereby escape from recognition (Heil, 2012). Therefore, and in analogy to the immune system of mammals, which detects endogenous danger signal to perceive the “wounded self” (Gallucci and Matzinger, 2001; Matzinger, 2002; Land and Messmer, 2012), it has been hypothesized that plants are able to perceive fragmented or delocalised own molecules as damage-associated molecular patterns (DAMPs), which enable “damaged-self recognition” (Heil, 2009, 2012).

Indeed, the application of leaf homogenate frequently triggers plant resistance responses, which can be directed against herbivores, pathogens or both (Heil, 2009). Leaf homogenate contains fragments of plant macromolecules such as oligosaccharides, oligogalacturonides, and peptides and its application to a leaf also causes the presence in the extracellular space of molecules such as sucrose, Adenosintriphosphate (ATP), and nucleic acids, which in the intact tissue would be localized within the cell (Heil, 2009). Most of these molecules are produced or released passively as soon as tissue is being disrupted: macromolecules become exposed to (lytic) enzymes from which they are compartimentally separated in the intact cell, and small molecules are released into the extracellular space. In principle, such molecules can serve as DAMPs. Indeed, extracellular sucrose is an important signaling molecule in plants (Sheen et al., 1999; Rolland et al., 2006; Bolouri Moghaddam and Van den Ende, 2013), extracellular ATP (eATP) has immunmodulatory effects in mammals (Schwiebert and Zsembery, 2003; Chen and Nuñez, 2010; Ivison et al., 2011; Zeiser et al., 2011), insects (Moreno-Garcia et al., 2014), plants (Demidchik et al., 2003; Kim et al., 2006; Roux and Steinebrunner, 2007; Heil et al., 2012; Choi et al., 2014; Tanaka et al., 2014) and fungi (Medina-Castellanos et al., data not shown), methanol is released in wounded plant tissues from cell wall pectins by the action of pectin methylesterase (Dorokhov et al., 2012), and larger fragments of the plant cell wall matrix (Doares et al., 1995; Creelman and Mullet, 1997; Stennis et al., 1998; Bergey et al., 1999; Narváez-Vásquez et al., 2005) or of the extracellular matrix of mammals (Schaefer, 2010; Zeiser et al., 2011) serve as indicators of the ongoing injury and trigger resistance responses.

Interestingly, authors who applied leaf homogenates obtained from other plants, or even from algae, frequently observed induced resistance to pathogens (Devaiah et al., 2009; Medeiros et al., 2009; Chandrashekhara et al., 2010; Akila et al., 2011; Ghazanfar et al., 2011; Jayaraman et al., 2011; Jithesh et al., 2012). By contrast, the application of conspecific leaf homogenates to leaves of cabbage (*Brassica oleracea*) or maize (*Zea mays*) plants caused the emission of herbivore-induced volatile organic compounds (HI-VOCs) (Turlings et al., 1993; Mattiacci et al., 1995). Similarly, applying conspecific leaf homogenate to wild lima bean (*P. lunatus*) induced the secretion of extrafloral nectar (EFN), enhanced the levels of endogenous jasmonic acid (JA), and caused an overall transcriptomic response that was very similar to the response to exogenous JA application (Heil et al., 2012). JA is the central hormone in the octadecanoid signaling pathway that controls multiple induced plant defense traits (including EFN and HI-VOCs) to chewing herbivores (Wasternack, 2007; Pieterse et al., 2009; Thaler et al., 2012). HI-VOCs play diverse roles in the resistance of plants, the most commonly reported one being the attraction of predators and parasitoids as a means of indirect defense against herbivores (Dicke and Baldwin, 2010). EFN is a taxonomically widespread, HI attractant of ants, wasps, and other predators and serves the indirect defense of plants against herbivores (Heil, 2008, 2011). Hence, it appears that leaf homogenates obtained from conspecific plants usually induce the resistance to herbivores, whereas homogenates from heterospecific plants more frequently induce the resistance to pathogens.

**FIGURE 1:**
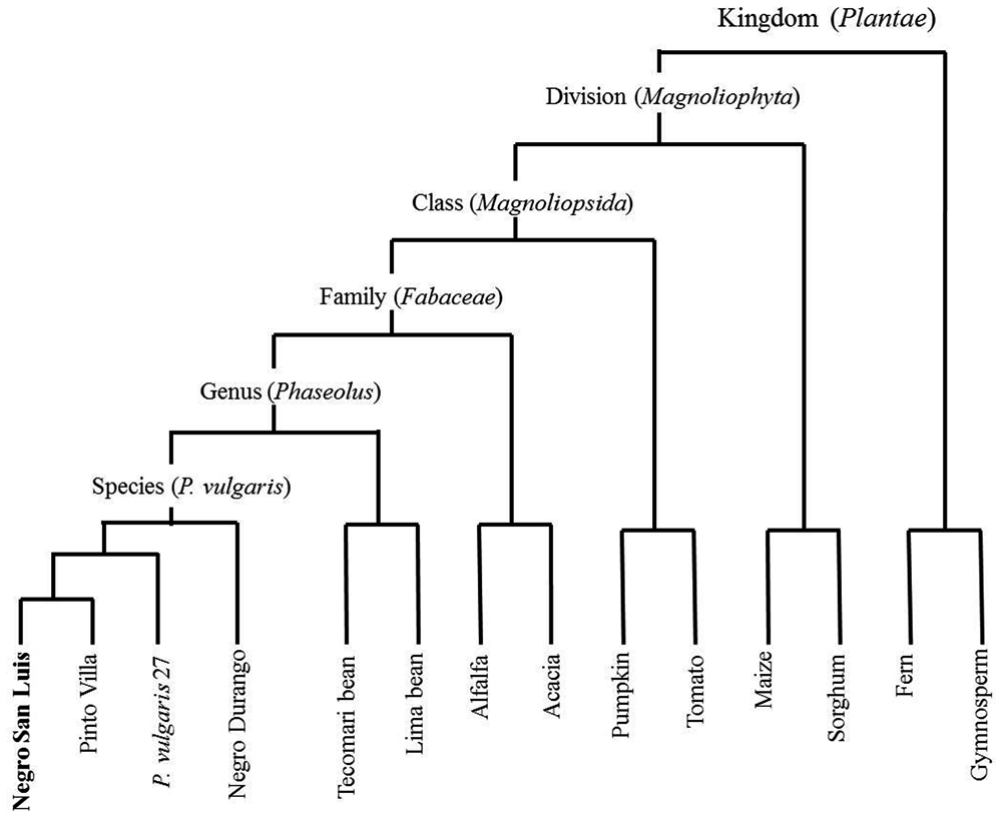
**Phylogenetic relations to the receiver, *Phaseolus vulgaris* cultivar “Negro San Luis” (NSL), of the plant species used for the preparation of leaf homogenates.** The relative phylogenetic distances among the different accessions within the species *P. vulgaris* were assessed using AFLPs (data not shown) whereas relations among species are depicted according to public information (URL http://www.ncbi.nlm.nih.gov/taxonomy/).

In the present study we used cultivated common bean (*P. vulgaris*, cultivar Negro San Luis, “NSL”; Fabaceae: Faboideae) to investigate whether leaf homogenates from species with different degrees of relatedness to the receiver plant (**Figure 1**) cause different responses. NSL-plants were treated with homogenate obtained from the same cultivar, the common bean cultivars, Pinto Villa and Negro Durango, a wild accession of common bean, and from Tecomari bean (*P. coccineus*), Lima bean (*P. lunatus*), Alfalfa (*Medicago sativa*; all: Fabaceae: Faboideae), an Acacia (*Acacia farnesiana*; Fabaceae: Magnolioidea), pumpkin (*Cucurbita maxima*; Cucurbitaceae), tomato (*Solanum lycopersicum*; Solanaceae), maize and sorghum (*Sorghum spp*.; both: Poaceae), a gymnosperm (*Pinus arizonica*) and a fern (*Adiantum aleuticum*). We also exposed leaves to the headspace of leaf homogenates to determine whether VOCs contribute to a putative induction event. The formation of ROS was quantified as an early marker of general resistance responses and EFN secretion was quantified as a late marker of the octadecanoid-dependent responses to herbivores. We also challenged the plants with the biotrophic bacterial pathogen *Pseudomonas syringae*, to quantify the induced resistance to pathogens. With these experiments we aimed to understand whether resistance induction after the application of leaf homogenate depends only on universal DAMPs such as extracellular sucrose or eATP, or whether a more specific blend of DAMPs allows for true “damaged-self” recognition.

## MATERIALS AND METHODS

### BIOLOGICAL MATERIAL AND GROWING CONDITIONS

For all experiments, we used 4 week-old common bean plants as receivers (cultivar NSL; seeds were obtained from the national germplasm collection at INIFAP, Celaya, GTO, México). The plants were grown under greenhouse conditions, watered *ad libitum* and fertilized weekly with a commercial fertilizer (Ferviafol 20-30-10®;, Agroquímicos Rivas S.A. de C.V., Celaya, GTO, México). All experiments were conducted under greenhouse conditions (temperature of 20°C at night and an average of 29°C during the day, natural light regime). For the preparation of leaf homogenates, the common bean cultivars, Pinto Villa and Negro Durango, a wild accession of common bean (accession 27) and *P. coccineus* were obtained from INIFAP Celaya. *P. lunatus* seeds were collected in a wild population 5 km west from Puerto Escondido, state of Oaxaca in Southern Mexico (∼15°55′ N and 097°09′ W). *M. sativa*, *C. maxima*, *Lycopersicum esculentum*, *Z. mays,* and *Sorghum sp*. plants were collected from croplands near CINVESTAV (Irapuato, GTO, México), *A. farnesiana* and *P. arizonica* from uncultivated areas near CINVESTAV, and *A. aleuticum* in Mary nursery garden (Irapuato, GTO, México). The rifampicin-resistant *P. syringae* pv. *syringae* strain 61 was provided by Dr. Choong-Min Ryu (KRIBB, Daejeon, South Korea).

### PREPARATION AND APPLICATION OF THE HOMOGENATES

The optimal conditions for the preparation and application of the leaf homogenate were determined in a series of preliminary experiments to define the best detergent (supplementary Figure 1), a suitable concentration of the homogenate (supplementary Figure 2), and to determine that a resting time of 2 h between the preparation of the homogenate and its application is required to obtain maximum induction effects (supplementary Figure 3). We also used preliminary experiments to define the optimum time for the determination of ROS (supplementary Figure 4), for the determination of the optimum plant age (supplementary Figure 5), to select Tween20® as the best detergent to use (supplementary Figure 6) and to optimize the grinding conditions (supplementary Figure 7). See supplementary Text 1 for a detailed description of all preliminary experiments. To prepare the homogenate, 1 g of fresh leaf material was weighed, lyophilized, and ground in 10 mL of a 0.05% (v/v) aqueous solution of Tween20®; (Sigma Aldrich) in a blender (Osterizer®; classic model: 450-10; Sunbeam Products, Owosso, MI, USA). Then, 20 mL of 0.05% Tween20®; were added, homogenized and the resulting homogenate was maintained at room temperature for 2 h. The resulting homogenate was applied with a soft brush on both sides of all the leaves of the treated plants until all surfaces were completely wet. The same amounts of a 0.05% (v/v) aqueous solution of Tween20®; were applied on the leaves of the control plants.

### RESPONSES IN EFN SECRETION TO DIFFERENT LEAF HOMOGENATES

The EFNs of NSL plants were washed by applying distilled water to remove all accumulated nectar and then treated with the different leaf homogenates or Tween20®; as described above. As a further control, plants were mechanically damaged with a needle (Heil et al., 2001, 2012). Six plants were used per treatment. Twenty-four hours later, EFN was quantified on the stipules of the three youngest leaves as the amount of secreted soluble solids, by measuring the volume with a graduated microcapillary tube and the concentration with a portable ATAGO®; refractometer (see Heil et al., 2000, 2001; for details).

### VISUALIZATION OF H_2_O_2_

Hydrogen peroxide was detected visually by staining with 3,3-diaminobenzidine (DAB), as described earlier (Thordal-Christensen et al., 1997), with some modifications. Entire trifoliate leaves were cut off at the base of the petiole at 2 h after treatment with the leaf homogenates (which was the time of maximum response, see supplementary Figure 4) and immersed in a solution of 1 mg mL^-1^ DAB (Sigma Aldrich) and 2-(*N*-morpholino) ethanesulfonic acid (MES) 10 mM (Sigma – Aldrich) that was adjusted with HCl to pH = 3.8. Leaves were kept in this solution under daylight and at room temperature for 7 h. Then, the leaves were transferred to a methanol – acetone (3:1) mixture and kept overnight with orbital agitation (100 rpm) to remove chlorophyll. Then, a second wash was performed by maintaining the leaves 1 h in methanol – acetone 3:1. Dark brown areas indicate the presence of H_2_O_2_. The leaves were stored at room temperature in 50% glycerol for 1 day to regain their flexibility and then were scanned (García-Neria and Rivera-Bustamante, 2011).

### QUANTIFICATION OF H_2_O_2_

To obtain quantitative values for the concentration of H_2_O_2_, we used the method as described by Choi et al. (2007), with some modifications. Two hours after the application of leaf homogenate, 10 circles of 1 cm diameter were punched out of each of three leaves collected from different plants, weighted, and suspended in 1 mL Milli-Q water. This suspension was kept stirring for 10 min and then centrifuged at 12.000 *g* for 15 min. Of the supernatant, 10 μL were mixed with 90 μL of substrate solution containing ferrous iron and xylenol orange (Hydrogen Peroxide Assay Kit, National Diagnostics, Atlanta, GA, USA). Blanks were prepared with Milli-Q water instead of the sample. The mixture was incubated for 30 min at room temperature and the absorbance was measured at 560 nm in a microplate reader (Synergy 2, BioTek Instruments Inc, Winooski, VT, USA) and was compared to a calibration curve for which we used H_2_O_2_ at 0–250 nmol mL^-1^.

### RESISTANCE TO *Pseudomonas syringae*

In order to test for induced resistance to a pathogenic bacterium, we used as subset of six homogenates that were selected based on their capacity to induce EFN secretion: NSL and *P. vulgaris* 27 leaf homogenates were used as strong inducers, Tecomari bean and lima bean leaf homogenates were used as medium inducers, and *A. farnesiana* and maize were used as two sources of homogenates that caused no detectable induction of EFN secretion. Each type of homogenate was applied to 10 plants. Control plants were treated either with Tween20®; (0.05 % v/v) or with distilled water (three groups of *n* = 5 plants for each water and Tween20®;). Five minutes after the application of the homogenate, *n* = 5 randomly selected plants per treatment (or the respective controls) were inoculated by spraying 10 mL per plant of a suspension of *P. syringae* (at 1 × 10^7^ cells mL^-1^, determined as optical density = 0.06 at 600 nm 5 in a GENESYS^TM^ 20 spectrophotometer; Thermo Fisher Scientific Inc, NY, USA). The remaining plants were inoculated in the same way 24 h after the application of homogenates and the third group of control plants was mock-inoculated.

In order to quantify infection levels, one leaf from each of six plants per treatment was collected 8 days after inoculation, weighed and ground in a mortar with approximately 500 μL of sterile distilled water. The resulting liquid was decanted and collected in a tube and was gaged to 1.5 mL with sterile distilled water. Dilutions 1:10, 1:100 and 1:1000 were prepared from each sample and 20 μL of each dilution were plated on KB medium [B medium as described by King et al. (1954) with rifampicin (100 μg mL^-1^; Sigma Aldrich)]. The colony forming units (CFUs) were counted 2 days later.

Putative direct effects of the homogenates against *P. syringae* were tested by plating 100 μL of each of the homogenate or of the control treatments (Tween20®; at 0.05% or sterile distilled water, *n* = 4 repetitions per type of homogenate) on Petri dishes with KB medium with rifampicin. After 5 min, 20 μL of a 1:10 1:100, 1:1 000, or 1:10,000 v/v dilution of 1 × 10^7^ cells mL^-1^ *Pseudomonas* suspension were spread on the same plates. A group of *n* = 4 plates for each homogenate and the control treatment were left without inoculation. The CFU in each Petri dish were counted 2 days later.

### EFFECT OF VOLATILE COMPOUNDS ON EFN SECRETION

We aimed at investigating whether VOCs emitted from the leaf homogenates might play a role in the induction process. For that purpose, six groups of *n* = 6 plants each were treated as follows: (1) mechanical damage (MD) and water; (2) only water; (3) fresh leaf homogenate applied directly on the leaves (see above); (4) fresh leaf homogenate brought close to the leaves but without direct contact; (5) lyophilized leaf homogenate applied directly on the leaves; (6) lyophilized leaf homogenate brought close to the leaves but without direct contact. To bring homogenates close to the leaves with no direct contact (treatments 4 and 6), spoon-shaped aluminum containers were produced and 2 mL of homogenate was placed in each of them, positioned at ca 5 cm below each leaf. Both leaf and aluminum container were enclosed in a transparent bag (polypaper 15 × 25 cm; SEMAPlastic, León, GTO, México; see supplementary material) to maintain all volatile compounds in the headspace around the leaves. The secretion of EFN was quantified 24 h after starting these treatments, as described above.

### EFFECT OF eATP ON EFN SECRETION

Groups of seven NSL bean plants were treated with aqueous solutions of 0.05% Tween20®; containing different concentrations of ATP (1, 0.5, 0.1, and 0.04 mM; Heil et al., 2012). ATP solutions were applied as described for leaf homogenates 1 h after washing nectaries and the secreted EFN was quantified 24 h later as described above. Plants treated with NSL bean leaf homogenate and with MD served as positive controls, plants treated with 0.05 % Tween20®; or water served as negative controls.

### EFFECT OF HEATING THE HOMOGENATE ON EFN SECRETION

We wanted to test whether the EFN-inducing activity of the leaf homogenates is affected by heating the homogenate in order to deactivate the endogenous enzymes that might be involved in the formation of DAMPs. To this end, eight groups of each *n* = 5 NSL plants were treated as follows (all liquids were applied as described above for the fresh leaf homogenates): (1) Tween20®; added to homogenate, then 2 h resting time; (2) Tween20®; added to homogenate, then 2 h resting time, then boiled; (3) Tween20®; added to homogenate, then boiled, then 2 h resting time; (4) homogenate with water only, then 2 h resting time, then Tween20®; added directly before application; (5) homogenate with water only, then 2 h resting time, then boiled, then Tween20®; added directly before application. All the “boiled” homogenates were heated 3 min to 100°C and applied at room temperature. The EFN secretion was quantified 24 h later.

## RESULTS

**FIGURE 2:**
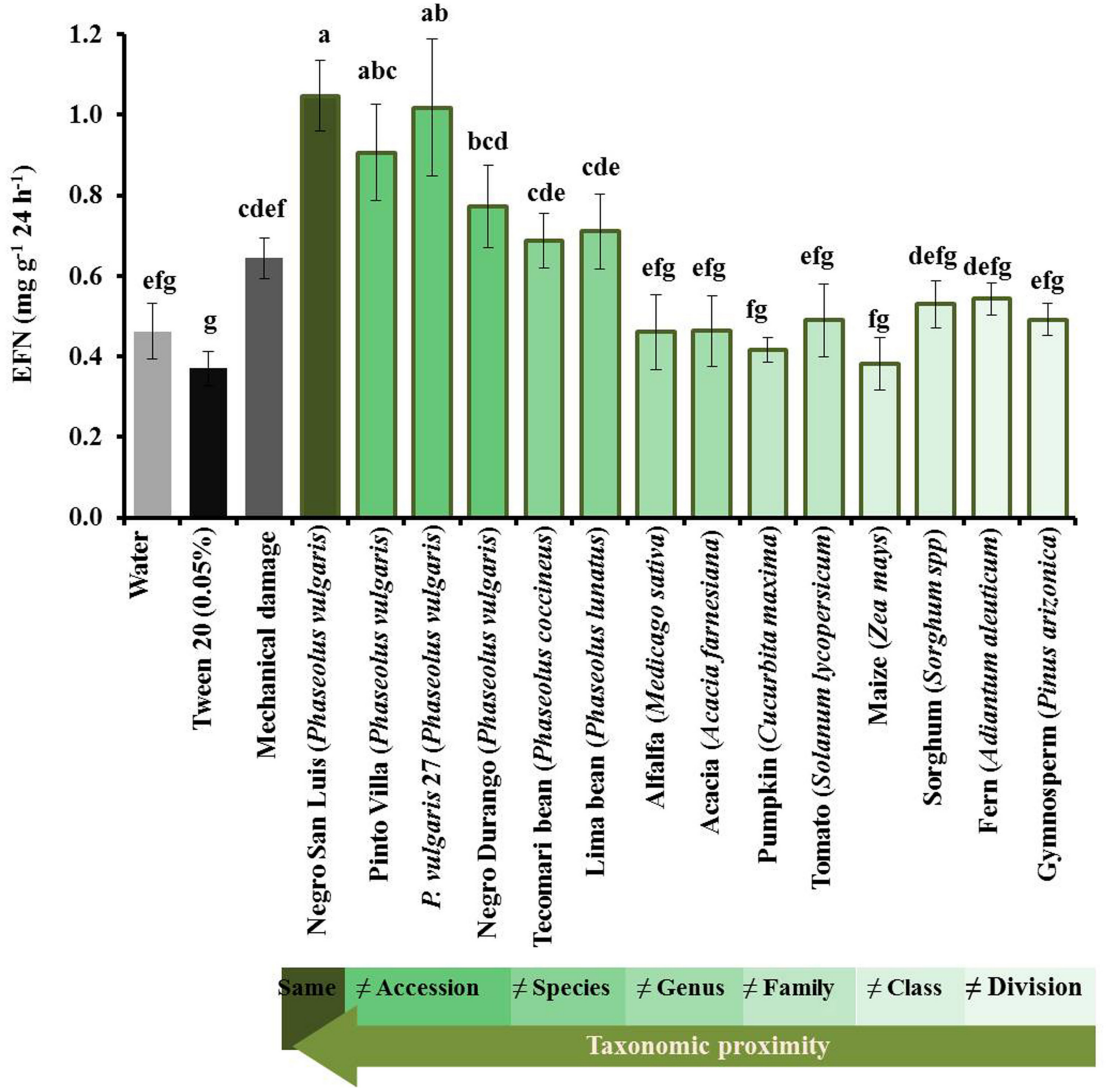
**Induction of extrafloral nectar (EFN) by leaf homogenates depends on phylogenetic distance.** The EFN secretion after applying different leaf homogenates on intact leaves is depicted as means ± SE of mg soluble solids per gram of dry leaf mass as quantified 24 h after treatment of *n* = 6 biological replicates per homogenate. Control plants were treated with water (light gray bars), Tween20 (black bars) or mechanically damaged (dark gray bars). The intensity of green of the bars indicates the relatedness among the receiver plant and the plants from which the homogenates were prepared. Different letters above bars indicate significant differences among treatments (univariate ANOVA and *post hoc* Tukey test: *p* < 0.05).

The secretion of EFN responded significantly to the application of leaf homogenate from closely related (i.e., congeneric) species, but not from other plants (**Figure 2**). Homogenate from NSL-plants and Pinto Villa-plants as well as from the wild common bean accession caused the strongest response. Lower, but still significant, induction of EFN was caused by the application of homogenate obtained from the common bean cultivar Negro Durango or from the two congeneric species, *P. coccineus* or *P. vulgaris*. Although these extracts induced a significant response over control levels, they did not induce EFN secretion more strongly than MD inflicted by a needle. By contrast, none of the non-bean species resulted in EFN secretion rates that differed significantly from the rates observed on the control plants.

**FIGURE 3:**
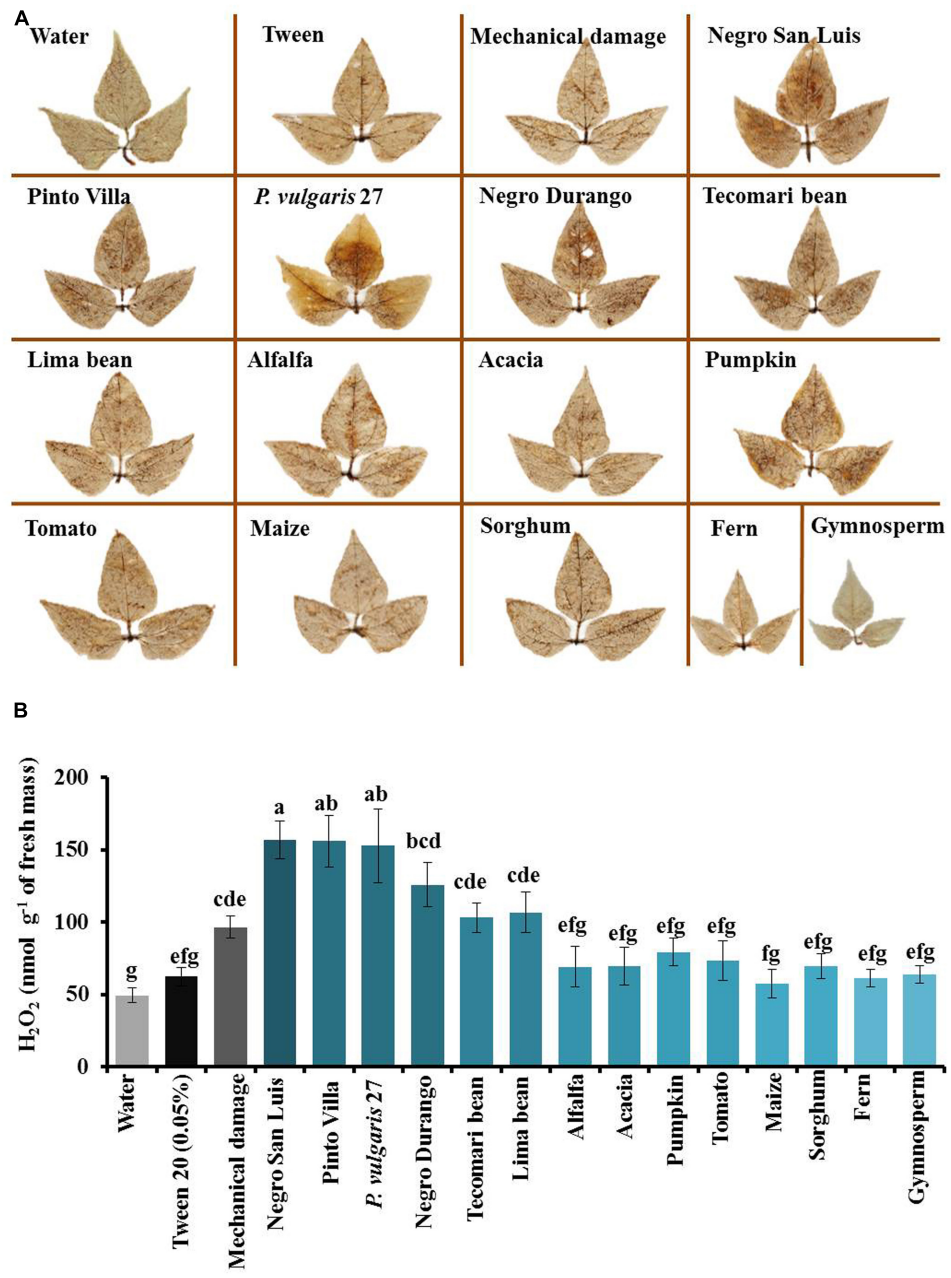
**Formation of hydrogen peroxide (H_**2**_O_**2**_) after application of leaf homogenates depends on phylogenetic distance. (A)** The presence of H_2_O_2_ was detected by staining with diaminobenzidine (DAB) 2 h after application of leaf homogenates. **(B)** Concentrations of H_2_O_2_ in nanomol per gram leaf fresh mass. The intensity of blue of the bars indicates the relatedness among the receiver plant and the plants from which the homogenates were prepared. Bars depict means ± SE of *n* = 3 biological replicates per homogenate and different letters above bars indicate significant differences among treatments (univariate ANOVA and *post hoc* Tukey test: *p* < 0.05).

Very similar patterns were observed in the formation of ROS (**Figure 3**). Punching holes with a needle caused local induction of ROS production and related leaf homogenates caused a strong presence of ROS at 2 h after application (**Figure 3A**, supplementary Figure 4). The quantification of H_2_O_2_ revealed that homogenates obtained from NSL-plants, Pinto Villa-plants or the wild common bean accession significantly enhanced the concentration of ROS in the leaves (**Figure 3B**). Lower responses were observed after applying homogenates from the cultivar Negro Durango, *P. coccineus* or *P. vulgaris*, and no significant response was caused by any of the non-bean species (**Figure 3B**).

**FIGURE 4:**
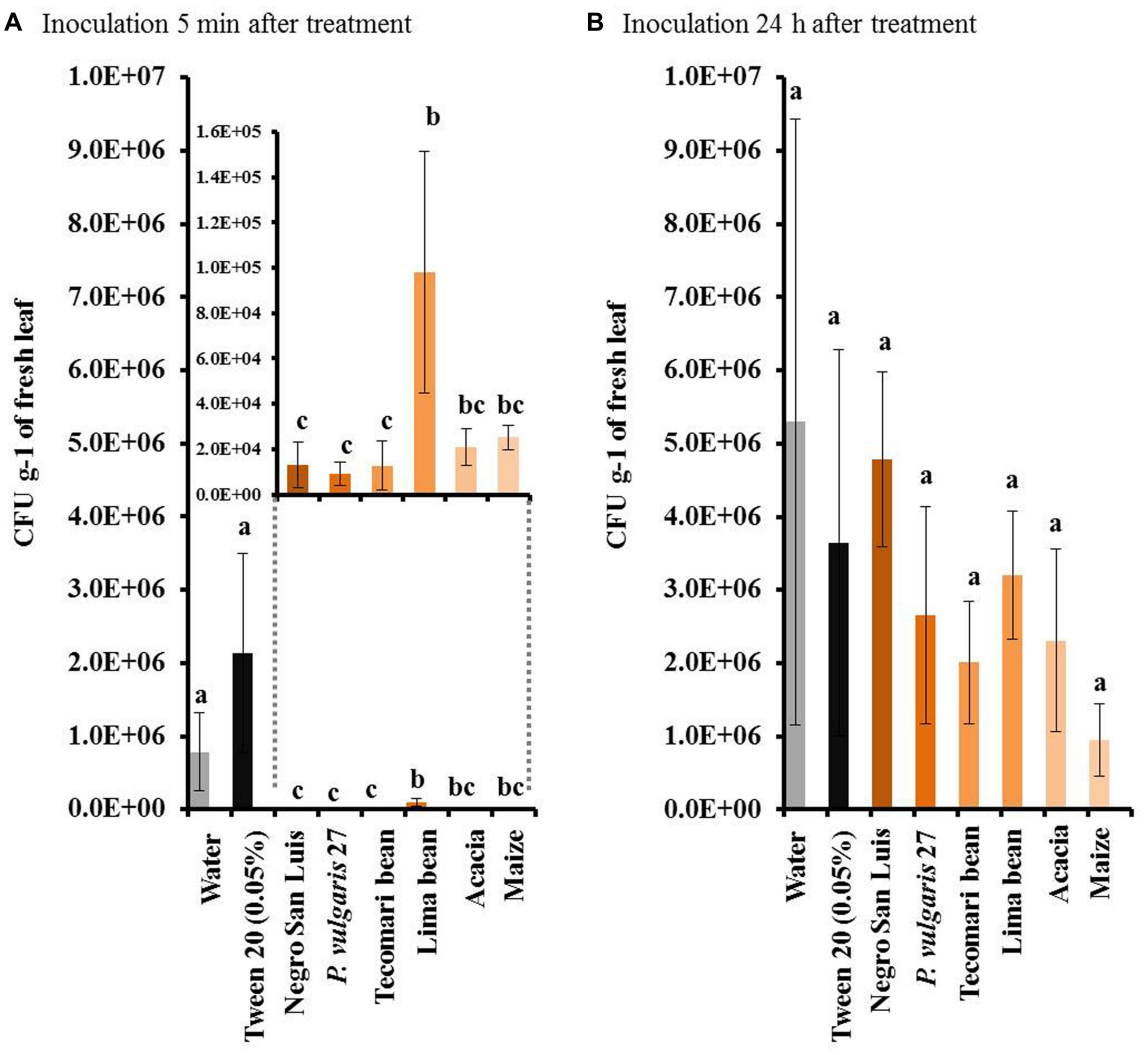
**Resistance to *Pseudomonas syringae* in plants treated with different leaf homogenates does not depend on phylogenetic distance.** We depict the number of colony forming units (CFUs) of *P. syringae* in leaves inoculated with the pathogen 5 min **(A)** or 24 h **(B)** after application of leaf homogenates. Bars indicate means ± SE of *n* = 6 biological replicates and different letters indicate significant differences among treatments (univariate ANOVA and *post hoc* Tukey test: *p* < 0.05).

**FIGURE 5:**
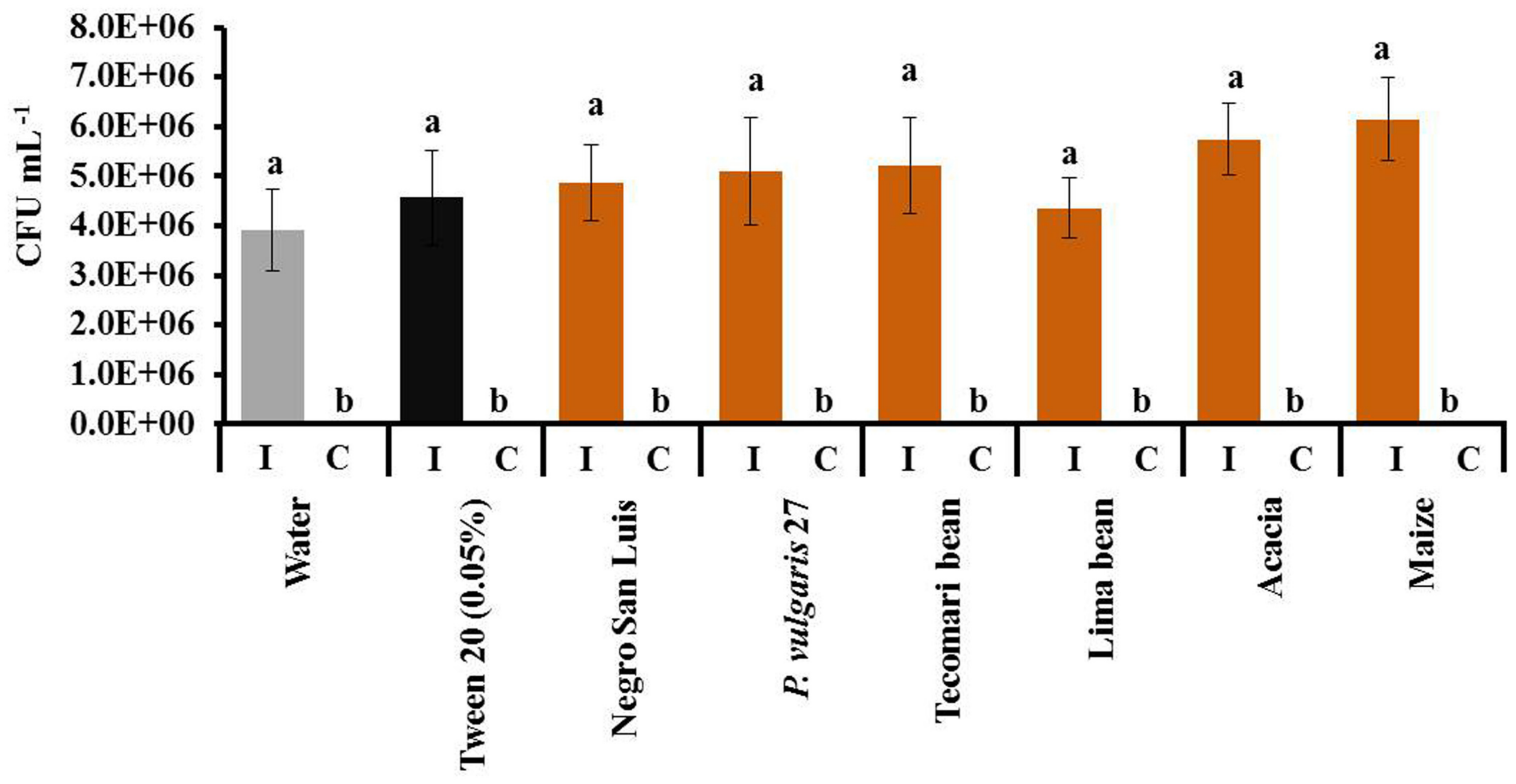
**Leaf homogenates do not directly inhibit *P. syringae*.** We depict numbers of CFUs 2 days after inoculating (I) the bacterium on Petri dishes that 5 min before had been prepared with sterile distilled water, Tween20^®^ or the indicated leaf homogenates. Bars indicate means ± SE of *n* = 4 repetitions and different letters indicate significant difference among treatments (univariate ANOVA and *post hoc* Tukey test: *p* < 0.05). Negative controls (C) were not inoculated with *P. syringae* to ensure that the leaf homogenates themselves did not carry bacteria.

All homogenates tested (NSL, wild accession, *P. coccineus*, *P. lunatus*, *A. farnesiana* and maize) caused significant resistance to the bacterium, *P. syringae*, when they were applied 5 min before challenging the plant with the bacterium (**Figure 4A**). By contrast, no enhanced resistance to the bacterium could be observed when the leaf homogenate had been applied 24 h before the challenging treatment (**Figure 4B**). In a Petri dish assay, the leaf homogenates had no direct inhibitory effects on bacterial growth when the KB-medium was pre-treated with one of these homogenates 5 min before applying the bacterial suspension (**Figure 5**).

**FIGURE 6:**
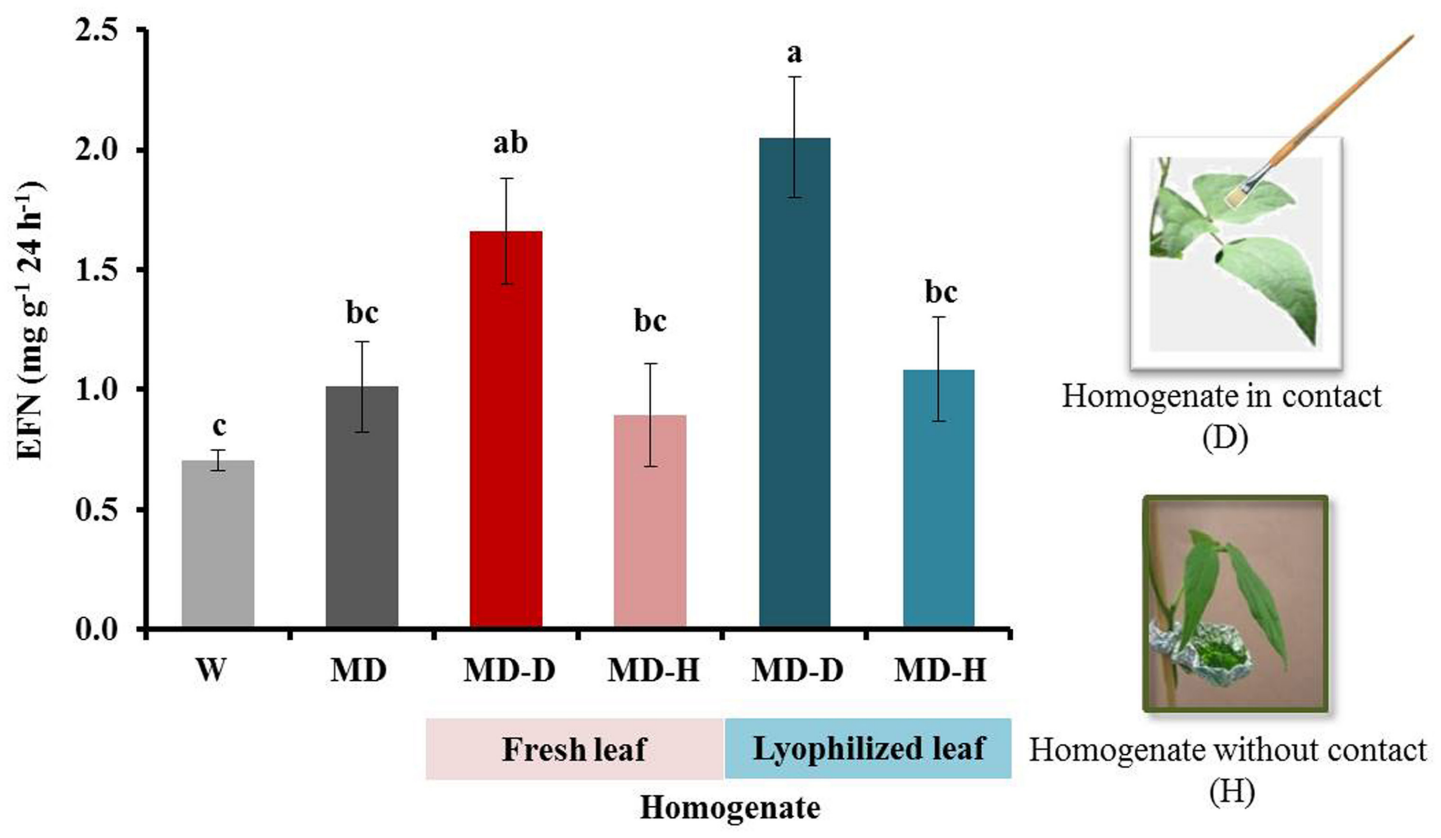
**Volatile organic compounds (VOCs) released from leaf homogenate do not induce EFN secretion.** EFN secretion (mg soluble solids per gram of dry leaf mass as quantified 24 h after treatment) was quantified on plants treated with mechanical damage (MD) and leaf homogenate of NSL plants obtained from fresh or lyophilized leaves. Leaves were directly treated with the homogenate (D) or had no direct contact, that is, they were only exposed to the headspace (H) of the homogenates. Controls were treated with water (W). Bars indicate the mean ± SE of *n* = 6 biological replicates and different letters indicate significant differences among treatments (univariate ANOVA and *post hoc* Tukey test: *p* < 0.05).

**FIGURE 7:**
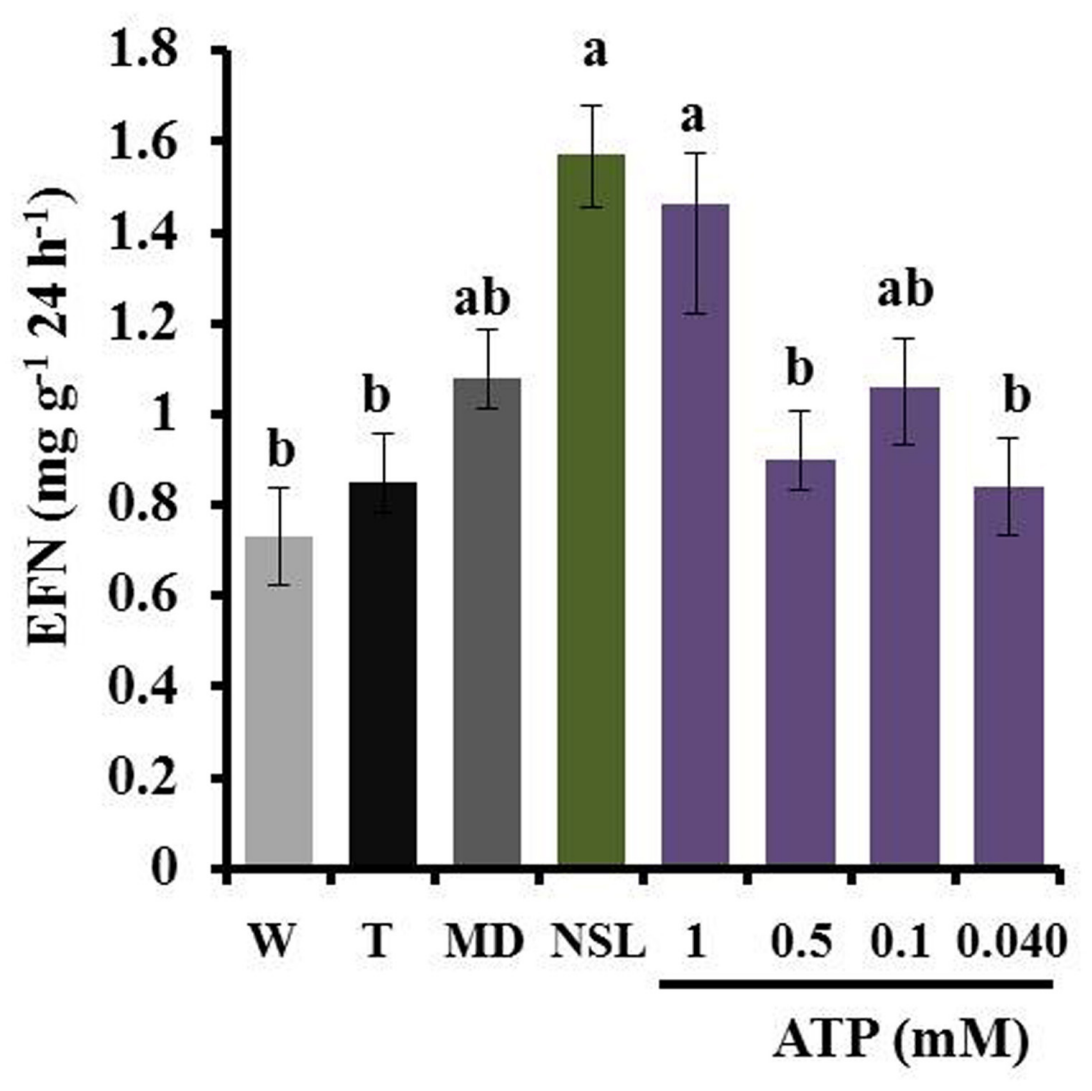
**Exogenous ATP at high concentrations induces EFN secretion.** EFN secretion (mg soluble solids per gram of dry leaf mass as quantified 24 h after treatment) was quantified on plants treated with different concentrations of exogenous ATP and compared to plants treated with NSL homogenate or MD as positive controls or with Tween20^®^ (T) or water (W) as negative controls. Bars indicate the mean ± SE of *n* = 7 biological replicates and different letters indicate significant differences among treatments (univariate ANOVA and *post hoc* Tukey test: *p* < 0.05).

When plants were exposed to leaf homogenate without any direct contact between homogenate and leaf surface, we could not detect any induction of EFN secretion. By contrast, the same homogenates caused an effect when they were directly applied on the leaves. Moreover, similar responses were observed after the use of fresh or lyophilized homogenates (**Figure 6**). The application of ATP induced EFN secretion only at a concentration of 1 mM but not at lower concentrations (0.5, 0.1, or 0.04 mM, see **Figure 7**).

**FIGURE 8:**
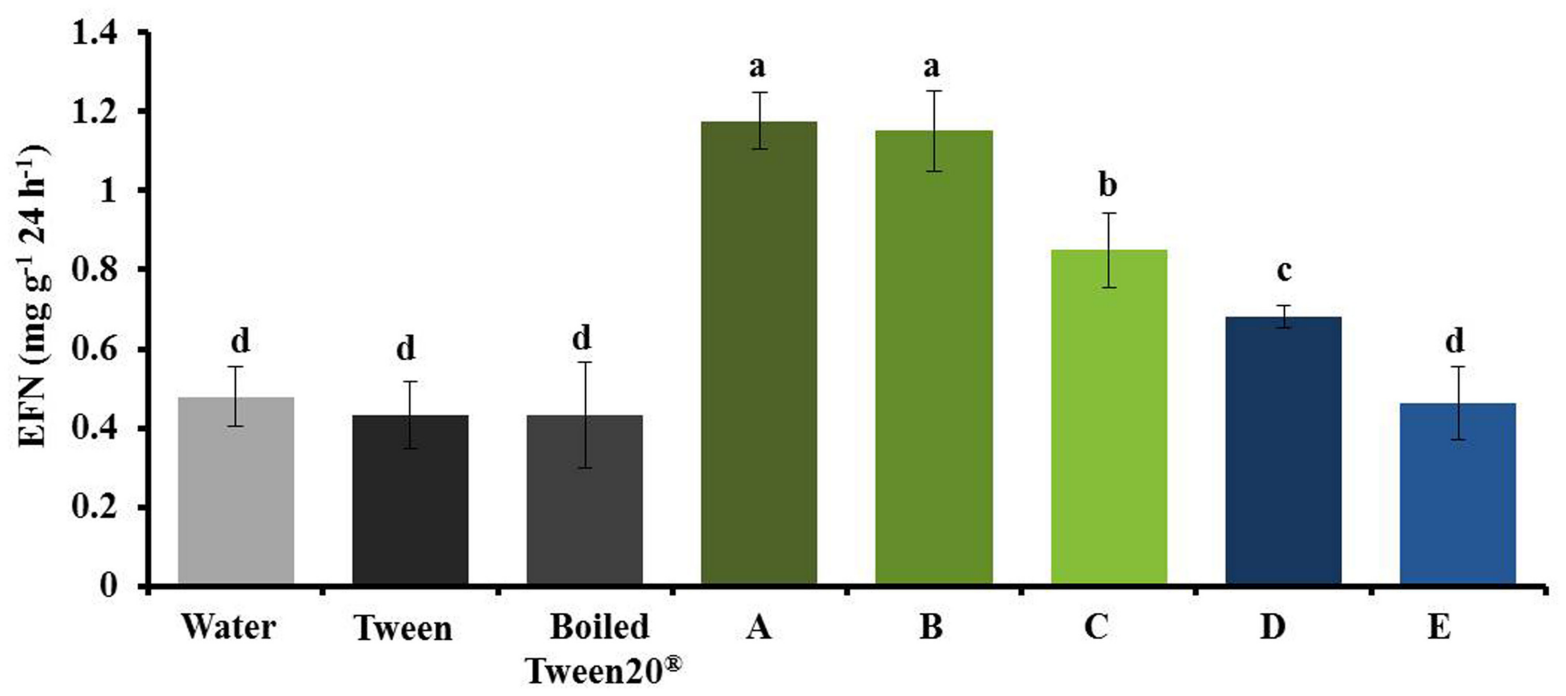
**Heat treatment of the homogenate before resting diminishes its effects on EFN secretion.** EFN secretion (mg soluble solids per gram of dry leaf mass as quantified 24 h after treatment) was quantified on plants treated with water, Tween20^®^, boiled Tween20^®^, or NSL leaf homogenate that was subjected to different temporal combinations of Tween20^®^ application, boiling (3 min at 100°C) and resting: **(A)** Tween20^®^ added to homogenate, then 2 h resting time; **(B)** Tween20^®^ added to homogenate, then 2 h resting time, then boiled; **(C)** Tween20^®^ added to homogenate, then boiled, then 2 h resting time; **(D)** homogenate with water only, then 2 h resting time, then Tween20^®^ added directly before application; **(E)** homogenate with water only, then 2 h resting time, then boiled, then Tween20^®^ added directly before application. Bars indicate the mean ± SE of *n* = 7 biological replicates and different letters above bars indicate significant differences among treatments (univariate ANOVA and *post hoc* Tukey test: *p* < 0.05).

During method establishment we had detected that the homogenates required at least 1 h resting time before application to elicit maximum responses (supplementary Figure 3) and that adding 0.05% Tween20®; enhanced the response of plants to a given homogenate (**Figure 8**, supplementary Figures 1 and 6), likely because Tween20®; facilitates the uptake of lipophilic compounds into the leaf tissue. However, Tween20®; enhanced the EFN-inducing effect of a homogenate even further when it was added to the homogenate before a resting time of 2 h (**Figure 8**). By contrast, no induction of EFN secretion was observed when the homogenate was heated for 3 min to 100°C before the 2 h of resting time, independent of whether or not Tween20®; had been added, whereas boiling the homogenate immediately before its application on the leaf did not diminish its EFN-inducing effect (**Figure 8**).

## DISCUSSION

Applying leaf homogenates obtained from bean plants on the leaves of common bean induced the formation of ROS after 2 h and the secretion of EFN over the following 24 h, and decreased the infection by the bacterial pathogen, *P. syringae*, over the following 2 days. No insects, pathogens, HAMPs, or MAMPs/PAMPS were used in any of the induction treatments. Thus, our results show that leaf homogenates contain DAMPs that can trigger both early and late resistance-related responses in intact, non-infected leaves. eATP induced EFN secretion in this and an earlier study (Heil, 2012) and in fact, eATP represents a universal DAMP that is active in organisms across the tree of life (Heil and Land, 2014). However, we observed a strong phylogenetic signal in the intensity of the formation of ROS and the induction of EFN by different homogenates: homogenates produced from leaves of the own cultivar and two genetically very similar common bean accessions (one cultivar and one wild accession) elicited the strongest response, a less related common bean cultivar and two other species of *Phaseolus* (*P. coccineus* and *P. lunatus*) elicited significant, but lower responses, and none of the non-bean species caused a significant induction of ROS formation or EFN secretion. We conclude that both responses are induced by a blend of DAMPs, rather than a single DAMP of wide taxonomic distribution, and that the entire blend of DAMPs that is released from the destroyed own cells allows plants to specifically detect the “damaged-self,” rather than allowing only for a general, diffuse “damaged plant” recognition. No phylogenetic effects were observed in the induced resistance to the pathogen: all homogenates reduced the rate of infection rate by *P. syringae* when they had been applied 5 min before inoculation. Because none of the leaf homogenates tested caused any detectable direct inhibitory effect on the growth of *P. syringae*, we conclude that the short-term inhibitory effect on *P. syringae* that we observed in homogenate-treated bean plants indicates an enhancement of pathogen resistance that was likely to be caused by a few, conserved DAMPs. In this context, the microflora that was associated to the plants from which the homogenates had been produced might have interfered with the observed resistance phenotype. However, no fungi or bacteria could be detected in the control Petri dishes that received only leaf homogenate but no *P. syringae* suspension, and no endophytic fungi or bacteria could be cultivated on standard media from bean plants that were cultivated under the same experimental conditions as used in the present study (data not shown). Thus, we assume that the inhibitory effects on *P. syringae* that we observed in the *in planta* assays were more likely to represent a transient induction of resistance in the receiver plant, rather than direct inhibitory effects of components (of plant or microbial origin) of the homogenate. However, putative resistance-inducing effects of endosymbiotic microorganisms cannot be excluded by now and require further investigation that will also have to consider non-culturable and, perhaps, intracellular endosymbionts.

The detailed nature of all DAMPs that are involved in this highly specific “damaged-self recognition” in plants will form the goal of future research efforts. We observed that heating the homogenates to 100°C for 3 min before the 2 h resting time strongly reduced the induction effect, which indicates that enzymatic activities and/or intact peptides or proteins are likely to be involved in the formation of the DAMPs. Since mixing the homogenate with Tween20®; 2 h before the application resulted in stronger induction effects than mixing the homogenate with Tween20®; directly before the application, we conclude that enzymatic activity during the 2 h of resting time might have helped with the formation of the full blend of DAMPs and that the disintegration of membranes by the detergent further contributed to DAMP formation, likely by exposing the endogenous enzymes to their substrates. Lyophilized homogenates were as effective as fresh homogenates, whereas no induction effect could be observed when leaves were only exposed to the headspace of the homogenates. Thus, it appears that VOCs did not play a significant role in the induction effects that we observed here, although they are well-known to induce or prime resistance responses in several plants, including bean (Engelberth et al., 2004; Heil and Kost, 2006; Kessler et al., 2006; Frost et al., 2007; Heil and Silva Bueno, 2007; Ton et al., 2007; Yi et al., 2009).

Plant resistance responses to biological enemies are orchestrated by two main signaling pathways, of which the salicylic acid (SA) pathway mainly controls responses to biotrophic pathogens, whereas ethylene/JA signaling mainly controls the resistance to chewing herbivores and necrotrophic pathogens (Pieterse and Dicke, 2007; Pieterse et al., 2009). In most plant species, these two pathways are subject to a negative crosstalk (Thaler et al., 2012). In our study, the formation of ROS and the secretion of EFN were positively correlated with each other, and both responded strongest to the same treatment. The formation of ROS is a widespread early response in plants and mammals to abiotic stress, invasion by pathogens or exposure to DAMPs (see references in Heil and Land, 2014), whereas the secretion of EFN serves the indirect defense of plants against insect herbivores (Heil, 2008). Thus, our results appear to contradict a major paradigm in induced plant defenses. However, the formation of ROS as an early event also been reported after herbivory in *Arabidopsis thaliana* (Beneloujaephajri et al., 2013) and sweet potato (Rajendran et al., 2014) or after sterile mechanical wounding in multiple plant species (Orozco-Cardenas and Ryan, 1999), and enhanced concentrations of ROS were observed after MD in the fungus, *Trichoderma atroviride* (Medina-Castellanos et al., data not shown). Thus, ROS might represent early signals in the response to injury in all multicellular organisms. Finally, responses that can be observed after mechanical injury in plants such as the release of green leaf volatiles (Heil and Karban, 2010; Dorokhov et al., 2012; Scala et al., 2013) or methanol (Dorokhov et al., 2012) and the synthesis of terpenoids such as limonene (Byun-McKay et al., 2006) all are directed against both pathogens and herbivores. Even serine proteinase inhibitors, which represent the first group of wound-inducible defensive proteins that have been discovered in plants (Green and Ryan, 1972), act against endopeptidases in both animals and microorganisms (Turra and Lorito, 2011).

Like all multicellular organisms, plants rely on an intact surface to prevent infection and desiccation. In the words of Komarova et al. (2014), “plant wounding is one of the conditions for pathogen entry.” Thus, as soon as a plant is damaged, it must seal the wound and prepare locally for infection. Applying leaf homogenates 5 min (but not 24 h) before challenging with *P. syringae* significantly decreased the levels of infection by this bacterium, and this effect depended much less on the detailed taxonomic nature of the homogenate than the induction of ROS development or EFN secretion. Because we observed no direct inhibitory effect of the homogenates on the bacterium in *in vitro* assays, we conclude that the application of leaf homogenate caused an unspecific and transient induction of the resistance in bean to pathogens, but no longer-lasting priming or induction of pathogen resistance, as it has been observed in VOC-exposed plants (Yi et al., 2009; Scala et al., 2013).

In summary, leaf homogenates contain a complex blend of DAMPs, which trigger early responses in plant tissues that are exposed to these DAMPs. These responses can be directed against both, pathogens and herbivores. An emerging general pattern appears to be that homogenates obtained from the same species trigger mainly responses against herbivores, whereas heterospecific leaf homogenates can enhance the resistance to pathogens. Many human “danger signals” play a dual role in resistance induction and act both as antimicrobial agents and as signals that enhance immunity-related responses in intact cells (Gallucci and Matzinger, 2001). Similarly, green leaf volatiles (Dorokhov et al., 2012; Scala et al., 2013), MeOH (Dorokhov et al., 2012) and wound-induced terpenoids such as limonene (Byun-McKay et al., 2006) have direct antimicrobial activity and also act as signals that induce the expression of defense-related genes in the surrounding tissues, distant parts of the same plant, and even in neighboring plants. The formation of ROS appears to play a central role in the responses in plants, fungi, and mammals to biotic and biotic stress and increasing evidence indicates their direct involvement in the perception of DAMPs in all these organisms. Future studies will have to identify the DAMPs are used by plants to perceive the “damaged-self” and to investigate which of these astonishing similarities and parallels between the ways in which plants and animals respond to injury represent the product of parallel evolution and which ones represent homologies.

## Conflict of Interest Statement

The authors declare that the research was conducted in the absence of any commercial or financial relationships that could be construed as a potential conflict of interest.
