# Supplementary material for: Damaged-self recognition in common bean (Phaseolus vulgaris) shows taxonomic specificity and triggers signaling via reactive oxygen species (ROS)
Source: Front Plant Sci. 2014 Oct 31;5:585. doi: 10.3389/fpls.2014.00585 (PMC4215620; doi:10.3389/fpls.2014.00585)
Supplement: Supplementary file 1 [file Data_Sheet_1.DOCX]

**Duran-Flores & Heil - Frontiers in Plant Science 2014 - Supplementary text**

**Selection of bean plant accession and plant age**

Three common bean accessions (Negro Durango, Negro Papaloapan and Negro San Luis) were selected, which under field conditions respond to exogenous application of jasmonic acid (JA) with an enhanced secretion of extrafloral nectar (EFN) ([Córdova-Campos, 2011](#_ENREF_1)). The plants were spray-treated with an aqueous solution of JA (1 mM) under greenhouse conditions (temperature of 20 ° C at night and an average of 29 ° C during the day, natural light regime). After 24 hours, the extrafloral nectar (EFN) was quantified on the stipules of the five youngest leaves as the amount of secreted soluble solids, by measuring the volume with a graduated microcapillary tube and the concentration with a portable ATAGO^®^ refractometer (see ([Heil et al., 2000](#_ENREF_4);[Heil et al., 2001](#_ENREF_6)) for details). We noted that Negro San Luis (NSL) secreted more EFN than Negro Durango or Negro Papaloapan and for this reason selected this cultivar for the present study. Quantifying EFN on JA-treated and water-treated NSL plants separately for each leaf revealed that the youngest leaf showed highest secretion rates (supplementary Figure 5). We also observed that 4 wk-old plants showed highest secretion rates and the strongest response to exogenous JA (supplementary Figure 5), for which reason 4 wk-old NSL plants were used for all consecutive experiments.

**Optimization of the preparation of leaf homogenates**

Earlier studies had shown that EFN secretion in lima bean responds to mechanical damage (punching holes with a needle) and the application of lima bean leaf homogenate to slightly damaged leaves {Heil, 2012 #4317}. For the present study, we wanted to optimize and standardize the preparation of leaf homogenates obtained from different species and aimed at eliminating the damage treatment that had been used in earlier studies.

**Selecting the detergent**

Several detergents were tested at a 0.5% v/v concentration (Methoxypolyethyleneglycol (MPEG), Triton^®^ x-100, Tween20^®^, sodium dodecyl sulfate (SDS), sodium deoxycholic acid (SDA), lithium dodecyl sulfate (LDS) and 3-[(3-Cholamidopropyl)dimethylammonio]-1-propanesulfonate hydrate (CHAPS)), which have been used in the application of herbicides or growth hormones ([Dybing and Currier, 1961](#_ENREF_2);[Petracek et al., 1998](#_ENREF_10);[Tamura et al., 2001](#_ENREF_11);[Knoche and M.J., 2006](#_ENREF_9)). Groups of three plants each were treated with NSL leaf homogenate prepared with the corresponding detergent. Plants treated with the respective detergent were used as negative controls, plants treated with mechanical damage and homogenate prepared in water as described above were used as positive control. At this concentration, all these detergents exceed the critical micelle concentration, so one would not expect a major effect at higher concentrations ([Jansen, 1964](#_ENREF_8);[Holloway et al., 1992](#_ENREF_7)). The secretion of EFN was quantified 24 h after starting these treatments. Based on the resulting secretion rates (supplementary Figure 1), Tween20^®^ was chosen because it caused no induction effect on its own and because its presence in the homogenate significantly enhanced the response of the plant.

Another test was conducted to compare various concentrations of Tween20^®^. Groups of five plants were treated with Negro San Luis leaf homogenate prepared using different Tween20^®^ concentrations (0, 0.001, 0.005, 0.01, 0.05 and 0.5% v/v). Plants treated only with the respective concentration of Tween20^®^ diluted in distilled water were used as negative controls, plants treated with mechanical damage plus aqueous homogenate were used as positive control. The secretion of EFN was quantified 24 h after starting these treatments. We found the strongest response at 0.05% v/v of Tween20^®^ (supplementary Figure 6). Thus, leaf homogenates with 0.05% Tween20^®^ cwere used in the consecutive experiment with no need to inflict any damage on the treated leaves.

**Optimizing the grinding technique for the preparation of leaf homogenates**

Groups of three plants each were treated with NSL leaf homogenate, which was produced using lyophilized or fresh leaves and varying grinding methods. Leaves were ground in a mortar with or without liquid nitrogen, or in a blender (Osterizer^®^ classic model: 450-10; Sunbeam Products, Owosso, MI, USA). In each case the leaf was ground either gently or strongly (which for the blender was: a pulse of 3 s for the 'gentle' or 1 min for the 'strong' homogenization) with 10 mL of Tween20^®^. The secretion of EFN was quantified 24 h after starting these treatments. We did not observe any significant difference between the different techniques provided that the leaf was strongly ground (supplementary Figure 7). For this reason, we decided to use the blender (1min homogenization time) as the most reproducible method for the preparation of all leaf homogenates.

**Optimizing the concentration of the foliar homogenate**

Groups of three plants each were treated with NSL leaf homogenate at various concentrations: 0, 1, 1.5, 3.3, 10, 20, 30, 40 % of fresh leaf w/v. To prepare the homogenate the corresponding amount of fresh leaf material was lyophilized and homogenized as described above. The plants treated with 0% of leaf were treated with only Tween20^®^ and served as control. The secretion of EFN was quantified 24 h after starting these treatments. We observed no response at 1.5 % concentration but a strong response at 3.3 %, which was not increased further when we used higher concentrations (supplementary Figure 2). For this reason 3% (w/v of leaf material was used for the preparation of all homogenates

**Effect of resting time of the homogenate on the induction of EFN**.

NSL leaf homogenates were left for different resting times (0, 1, 2, 12 or 24 h) at room temperature and then used or, after a resting time of 2 h, maintained frozen (at -80°C) for 1 week or 4 months before application. Plants treated with 0.05% v/v Tween20^®^ without any leaf homogenate were used as control. The secretion of EFN was quantified 24 h after starting these treatments. No difference in the response was found except when the homogenate was applied immediately after its preparation: 'fresh' homogenate did not cause any detectable induction effect (supplementary Figure 3).

**H_2_O_2_ production in induced leaves by foliar homogenate.**

The presence of hydrogen peroxide was first detected visually, at various times after application of foliar homogenate of Negro San Luis bean plants to the same line of bean or mechanically damaging leaves with a needle. The hydrogen peroxide was visualised by staining with 3,3-diaminobenzidine (see methods in main text). Leaves treated with Tween20® were used as controls. Maximum concentration of H2O2 was observed at 2 h after the treatment (supplementary Figure 4).

**References**

Córdova-Campos, O. (2011). *Inducción de defensa directa e indirecta en plantas de frijol (Phaseolus vulgaris L. y Phaseolus coccineus L.) y su relación con la domesticación* Masters, CINVESTAV - Irapuato.

Dybing, C.D., and Currier, H.B. (1961). Foliar penetration by chemicals. *Plant Physiology* 36**,** 169-174.

Heil, M. (2004). Induction of two indirect defences benefits Lima bean (*Phaseolus lunatus*, Fabaceae) in nature. *Journal of Ecology* 92**,** 527-536.

Heil, M., Fiala, B., Baumann, B., and Linsenmair, K.E. (2000). Temporal, spatial and biotic variations in extrafloral nectar secretion by *Macaranga tanarius*. *Functional Ecology* 14**,** 749-757.

Heil, M., Ibarra-Laclette, E., Adame-Álvarez, R.M., Martínez, O., Ramirez-Chávez, E., Molina-Torres, J., and Herrera-Estrella, L. (2012). How plants sense wounds: damaged-self recognition is based on plant-derived elicitors and induces octadecanoid signaling. *PLoS ONE* 7**,** e30537.

Heil, M., Koch, T., Hilpert, A., Fiala, B., Boland, W., and Linsenmair, K.E. (2001). Extrafloral nectar production of the ant-associated plant, *Macaranga tanarius*, is an induced, indirect, defensive response elicited by jasmonic acid. *Proceedings of the National Academy of Sciences of the USA* 98**,** 1083-1088.

Holloway, P.J., Wong, W.C., Atridge, H.J., Seaman, D., and Perry, P.B. (1992). Effects of polyoxyethylene surfactants on uptake of ethirimol and diclobutrazol from suspension formulations applied to wheat leaves. *Pesticide Science* 34**,** 109-118.

Jansen, L.L. (1964). Enhancement of herbicide activity: Relation of structure of ethylene oxide ether-type nonionic surfactants to herbicide activity of water-soluble herbicides. *Journal of Agricultural and Food Chemistry* 12**,** 223-227.

Knoche, M., and M.J., B. (2006). Effect of Triton X-100 concentration on NAA penetration through the isolated tomato fruit cuticular membrane. *Crop Protection* 23**,** 141-146.

Petracek, P.D., Fader, R.G., Knoche, M., and Bukova, M.J. (1998). Surfactant-enhanced penetration of benzyladenine through isolated tomato fruit cuticular membranes. *Journal of Agricultural and Food Chemistry* 46**,** 2346-2352.

Tamura, H., Knoche, M., and Bukovac, M.J. (2001). Evidence for surfactant solubilization of plant epicuticular wax. *Journal of Agricultural and Food Chemistry* 49**,** 1809-1816.
