# Supplementary material for: Damaged-self recognition in common bean (Phaseolus vulgaris) shows taxonomic specificity and triggers signaling via reactive oxygen species (ROS)
Source: Front Plant Sci. 2014 Oct 31;5:585. doi: 10.3389/fpls.2014.00585 (PMC4215620; doi:10.3389/fpls.2014.00585)
Supplement: Supplementary file 2 [file Data_Sheet_2.PDF]

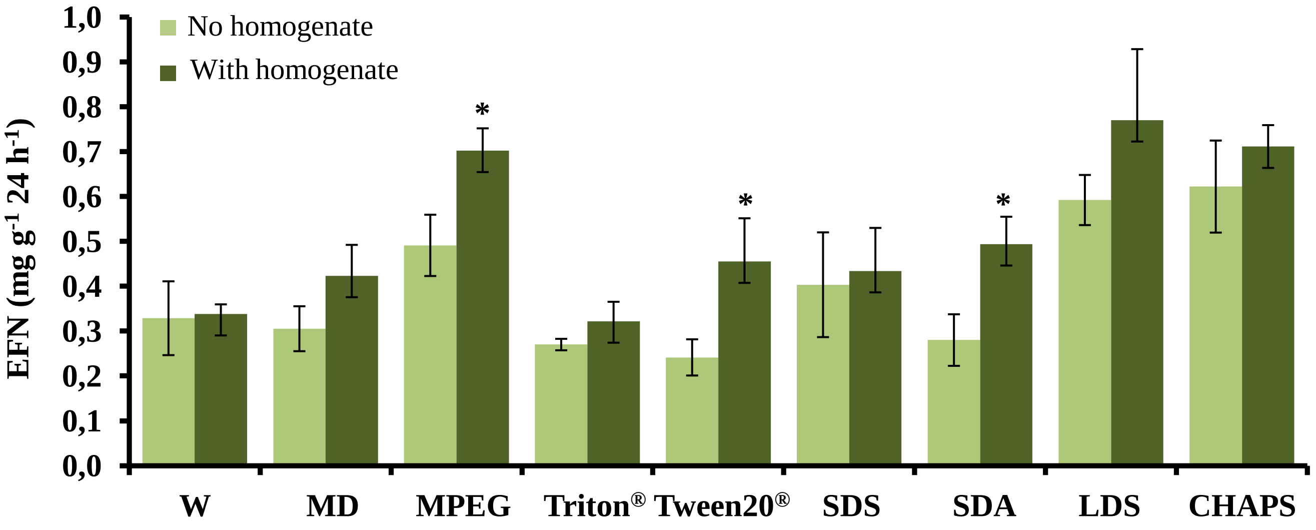

Supplementary Figure 1. Effect of different detergents in leaf homogenate on extrafloral nectar (EFN) secretion. The secretion of extrafloral nectar (mg soluble solids per gram of leaf dry mass 24 h after treatment) is depicted for intact leaves that were treated with NSL leaf homogenates (obtained from lyophilized leaves) that were prepared with various detergent agents at a 0.5% v/v concentration: Methoxypolyethyleneglycol (MPEG), Triton® x-100, Tween20®, sodium dodecyl sulfate (SDS), sodium deoxycholic acid (SDA), lithium dodecyl sulfate (LDS) and 3-[(3-Cholamidopropyl) dimethylammonio]-1-propanesulfonate hydrate (CHAPS). Control groups were only treated with the respective detergent that contained no homogenate, water (W) or mechanical damage (MD). Bars indicate the mean ± SE of n = 3 biological replicates and different letters indicate significant differences among treatments (Tukey test: $p<0.05$).

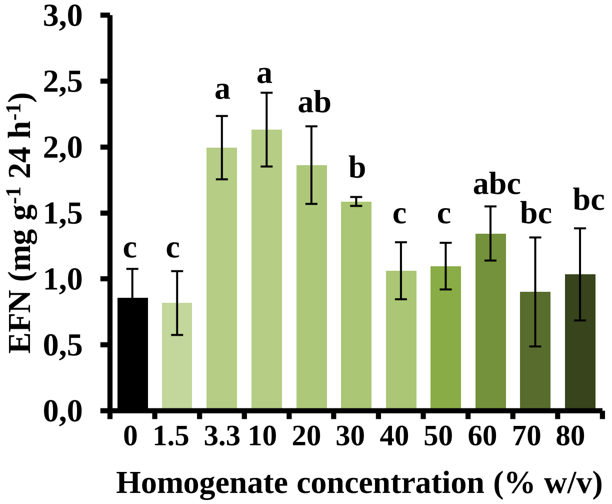

Supplementary Figure 2. Effects of different concentrations of foliar homogenate on extrafloral nectar (EFN) secretion. The secretion of extrafloral nectar (mg soluble solids per gram of leaf dry mass 24 h after treatment) is depicted for intact leaves that were treated with NSL leaf homogenate (obtained from lyophilized leaves and ground in an Osterizer®) prepared with 0.05% Tween20® and at various concentrations of foliar mass (0, 1.5, 3, 10, 20, 30, 40, 50, 60, 70 and 80% of fresh mass/v). Bars indicate the mean ± SE of n = 3 biological replicates and different letters indicate significant differences among treatments (Tukey test: $p<0.05$).

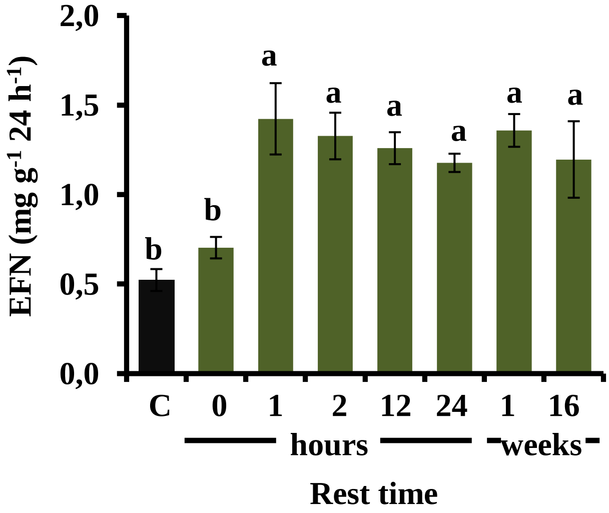

Supplementary Figure 3. Effect of resting time of the homogenate on the induction of EFN. The secretion of extrafloral nectar (mg soluble solids per gram of leaf dry mass 24 h after treatment) is depicted for intact leaves that were treated with NSL leaf homogenate that was allowed to rest over different times (0, 1, 2, 12 and 24 h at room temperature, and 1 week or 4 months kept frozen after a resting time of 2 h) before application. The control group (C) is formed by plants treated with fresh 0.05% v/v Tween20® without any leaf homogenate. Bars indicate the mean ± SE of n = 3 biological replicates and different letters indicate significant differences among treatments (Tukey test: $p<0.05$).

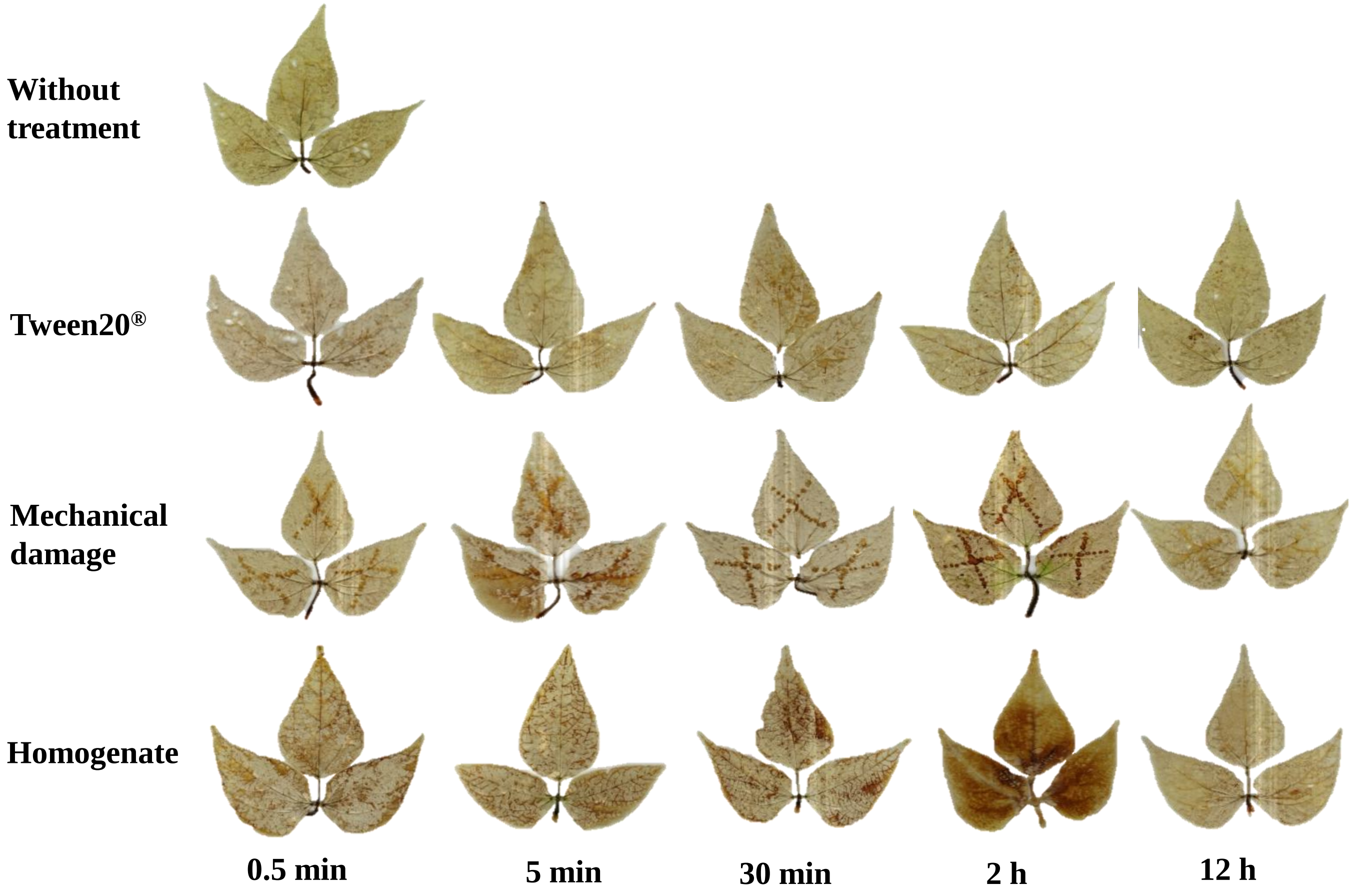

Supplementary Figure 4. Formation of H_2O_2 after mechanical damage and treatment with leaf homogenate. The presence of hydrogen peroxide was visualized at various times after punching holes with a needle or the application of NSL leaf homogenate, by staining with diaminobenzidine (DAB). H_2O_2 can be seen as dark brown spots.

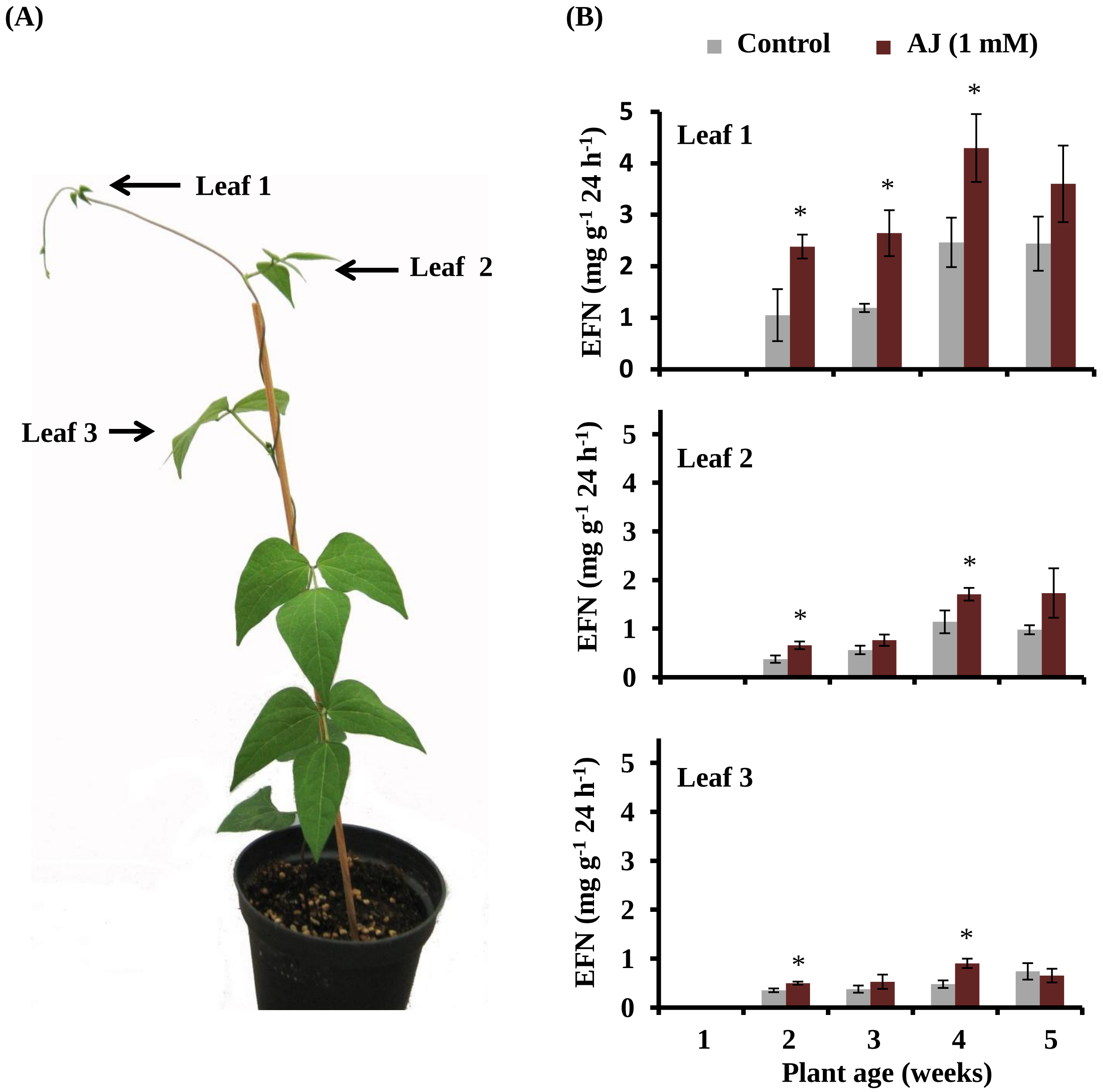

Supplementary Figure 5. EFN secretion by common bean depends on leaf age and plant age. (A) Definition of leaf numbers from 1 = youngest to 3 = oldest leaf used. **(B)** The secretion of extrafloral nectar (mg soluble solids per gram of leaf dry mass 24 h after treatment) is depicted for intact and individual leaves that were treated (control plants: light bars, plants treated with JA: dark brown bars) and sepearately for 1-5 wk of plant age. Bars indicate the mean ± SE of n = 5 biological replicates, asterisks indicate significant effects of JA treatment (Student t- test: $p<0.05$).

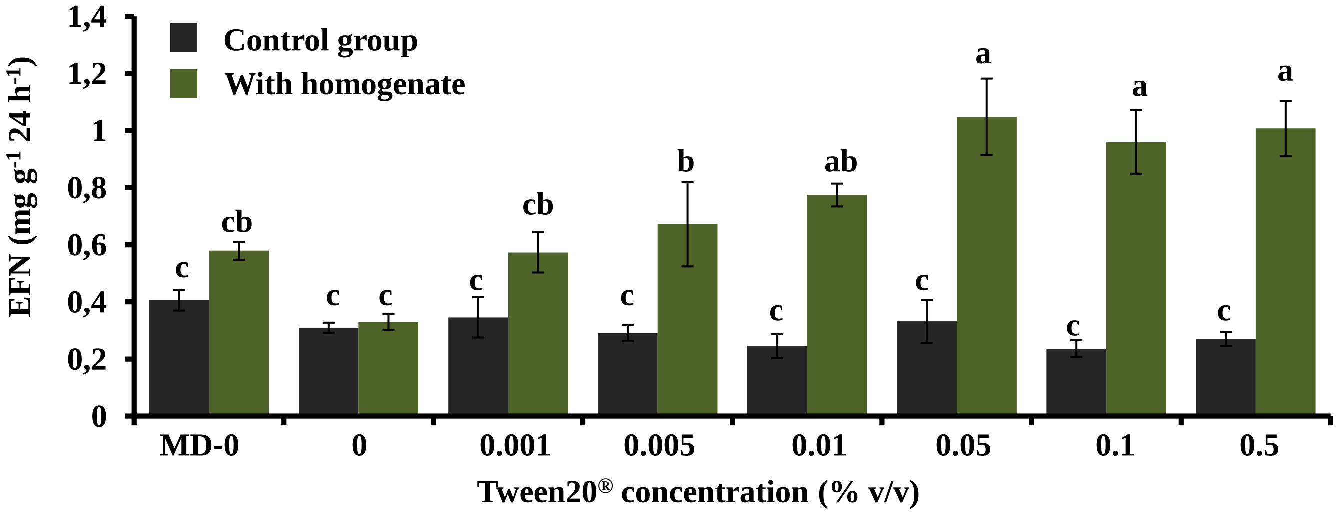

Supplementary Figure 6. Effects of different concentrations of Tween20® on extrafloral nectar (EFN) secretion. The secretion of extrafloral nectar (mg soluble solids per gram of leaf dry mass 24 h after treatment) is depicted for intact leaves that were treated with NSL leaf homogenate (obtained from lyophilized leaves) that were prepared using different concentrations (0 , 0.001, 0.005, 0.01, 0.05 and 0.5% v/v) of Tween20®. Controls were treated only with the respective concentration of Tween20® diluted in distilled water (black bars) or mechanically damaged. Tween20® treatments were compared with the application of water (0 % of Tween20®) on mechanically damaged plants and with plants mechanically damaged plus water (MD-0). Bars indicate the mean ± SE of n = 5 biological replicates and different letters indicate significant differences among treatments (Tukey test: $p<0.05$).

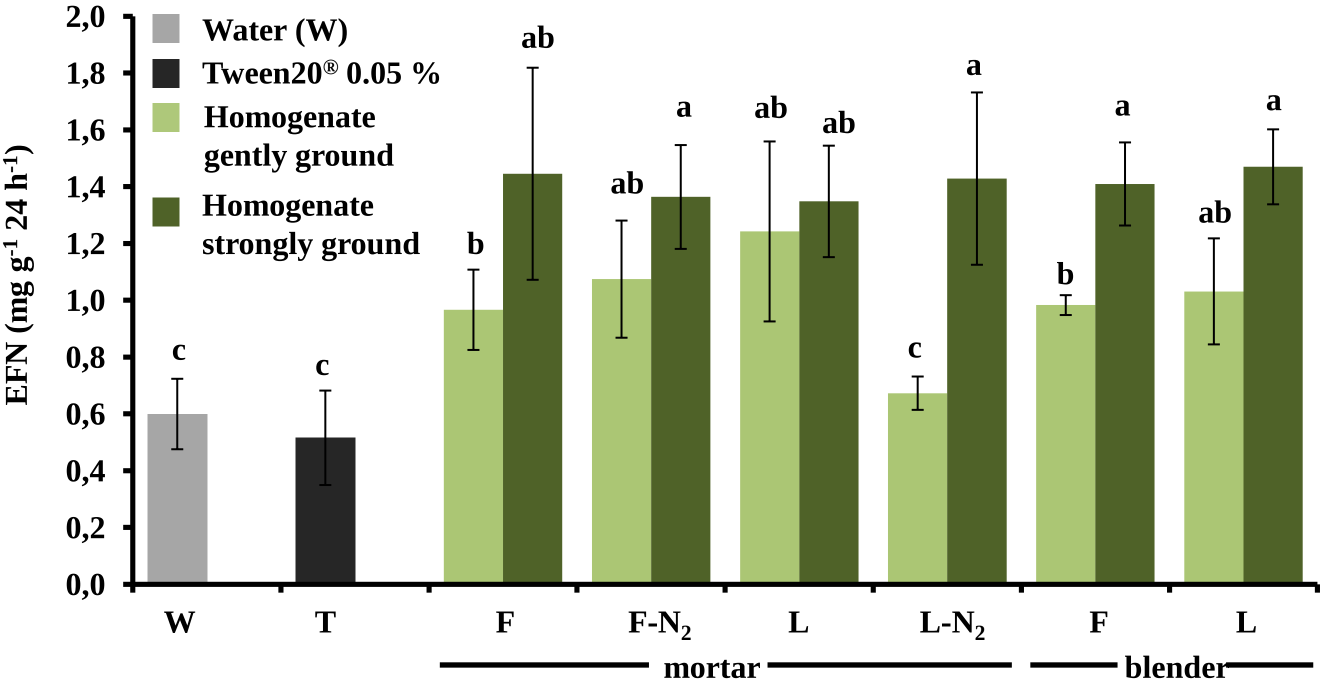

Supplementary Figure 7. Effect of the technique and intensity of grinding on EFN secretion. The secretion of extrafloral nectar (mg soluble solids per gram of leaf dry mass 24 h after treatment) is depicted for intact leaves that were treated with NSL leaf homogenate (obtained from lyophilized [L] or fresh [F] leaves) with 0.05% v/v Tween20®. The homogenates were prepared using varying grinding method (ground in mortar or blender, with or without liquid nitrogen, N_2). Water (W) or Tween20® treatments were used as controls. Bars indicate the mean ± SE of n = 3 biological replicates and different letters indicate significant differences among treatments (Tukey test: $p<0.05$).
